# Roles of Non-Coding RNAs in Normal Human Brain Development, Brain Tumor, and Neuropsychiatric Disorders

**DOI:** 10.3390/ncrna5020036

**Published:** 2019-04-30

**Authors:** Jun-Hua Nie, Tian-Xiang Li, Xiao-Qin Zhang, Jia Liu

**Affiliations:** School of Medicine, South China University of Technology (SCUT), Guangzhou 510006, China; mcnie@mail.scut.edu.cn (J.-H.N.); 201730690183@mail.scut.edu.cn (T.-X.L.)

**Keywords:** non-coding RNA, glioma, brain function, neuropsychiatric disease, miRNA, lncRNA, circRNA

## Abstract

One of modern biology’s great surprises is that the human genome encodes only ~20,000 protein-coding genes, which represents less than 2% of the total genome sequence, and the majority of them are transcribed into non-coding RNAs (ncRNAs). Increasing evidence has shown that ncRNAs, including miRNAs, long non-coding RNAs (lncRNAs), and circular RNAs (circRNAs), play important roles in regulating a wide range of biological processes of the human brain. They not only regulate the pathogenesis of brain tumors, but also the development of neuropsychiatric diseases. This review provides an integrated overview of the roles of ncRNAs in normal human brain function, brain tumor development, and neuropsychiatric disease. We discussed the functions and molecular mechanisms of miRNAs, lncRNAs, and circRNAs in normal brain function and glioma, respectively, including those in exosome vesicles that can act as a molecular information carrier. We also discussed the regulatory roles of ncRNAs in the development of neuropsychiatric diseases. Lastly, we summarized the currently available platforms and tools that can be used for ncRNA identification and functional exploration in human diseases. This study will provide comprehensive insights for the roles of ncRNAs in human brain function and disease.

## 1. Introduction

The human brain is a huge complex organ, composed of many different functional areas. The functions of each specific brain area are governed strictly by the appropriate expression of certain regulatory genes [1,2,3]. The alterations of these genes may not only result in psychiatric conditions, but also neurological disorders. Exploring the dysregulations of gene transcription and expression as well as the associated signaling pathways in the human brain in an integrated way, therefore, will help understand the complex regulating mechanisms of brain function and disease. 

Gliomas account for the great majority of primary brain tumors [4]. They can be further categorized into low-grade and high-grade (also called malignant tumor) tumors based on their malignancy degree [5]. Despite the development of multimodal and aggressive treatments that include surgical resection, local radiotherapy, and systemic chemotherapy, patient outcomes for the malignant glioma remain unsatisfactory in past decades [6,7]. To improve the treatment efficacy, a better understanding of glioma pathogenesis at molecular levels is urgently needed. 

It is very clear that non-coding RNAs (ncRNAs), including miRNAs, lnRNAs, and circRNAs, play important roles in brain function and disease [8,9,10]. They take the major part of human transcriptome, and engage in diverse structural, functional, and regulatory activities in the human brain. It has been reported that ncRNAs are dynamically expressed in the human brain with precisely regulated temporal and spatial expression patterns and mediate a broad spectrum of biological processes [1,2,3,8,9,10,11]. The dysregulation of ncRNAs may not only lead to disorders in brain function, but also tumorigenesis and neuropsychiatric diseases. 

While there have already been excellent reviews on each topic that will be discussed below, this review aimed to provide a general picture of the roles of non-coding RNAs in the human brain by summarizing the current available progress. We reviewed the growing evidence for the acting of miRNAs, lncRNAs, and circRNAs in normal human brain development, brain tumor development, and neuropsychiatric disease, respectively. We especially highlighted the regulatory mechanisms of ncRNAs that have been repeatedly reported in multiple independent studies. We also discussed the roles of extracellular ncRNAs in transferring regulatory information in glioma. Lastly, we summarized the publicly available tools and platforms that may help us explore the mechanism and regulatory functions of ncRNAs in human disease. 

## 2. Types and Functions of ncRNAs

**Figure 1:**
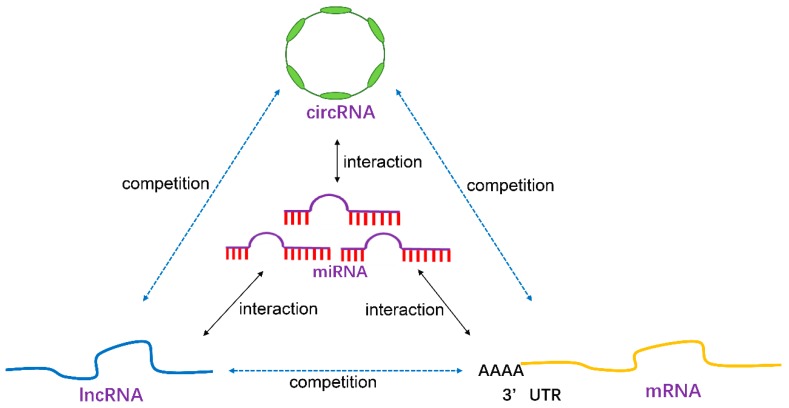
Interaction of different types of ncRNAs (miRNA, lncRNA, and circRNA).

NcRNAs contain multiple sub-classes, which mainly include transfer RNAs (tRNAs), ribosomal RNAs (rRNAs), miRNAs, piRNAs, snoRNAs, lncRNAs and the newly emerging circRNAs (Table 1). However, the functionally important types in human diseases usually refer to three types of them including miRNAs, lncRNAs, and circRNAs. They are also the focus of this current review. These three types of ncRNAs may not only work independently, but also interact with each other (Figure 1).

### 2.1. miRNAs

An miRNA is a small ncRNA molecule containing about 22 nucleotides. It mainly functions in RNA silencing and post-transcriptional repression of targeted gene expression [12,13]. However, it also has a “surprise” role that, under specific conditions, it can activate the translation of targeted mRNAs [14]. MiRNA has now been a well-studied ncRNA class and it can inhibit target mRNA or lncRNA gene expression by recognizing the complementary sequence in target RNA molecules, which are then silenced by cleavage into pieces [12,13]. The roles of miRNAs as key regulators of a wide variety of fundamental cellular processes, such as apoptosis, differentiation, proliferation, and cell cycle, and even clinical trials are increasingly recognized [13,15,16,17,18].

### 2.2. lncRNAs

LncRNAs are a class of linear mRNA-like RNA transcripts with at least 200 nucleotides in length, but cannot translate into protein. While the number of lncRNAs is much larger than that of miRNAs, their mechanisms for regulating gene expression and cellular function are not completely understood [19,20,21]. They were found to be involved in diverse biological processes through various mechanisms [19,20,21], and had especially abundant expression in the brain, which suggests their important and specific roles in brain function and disease [9,22]. The functional study for a small part of lncRNAs revealed that they had a broad range of functions, including roles in transcriptional and epigenetic mechanisms by recruiting transcription factors and chromatin-modifying complexes to specific genomic sites. This regulates alternative splicing and other post-transcriptional RNA modifications, nuclear–cytoplasmic shuttling, and translational control [19,20,21]. They can also act as competing endogenous RNAs (ceRNAs), which regulate other lncRNA or mRNA genes by competing for shared miRNAs, and form an lncRNA–miRNA–mRNA competing endogenous RNA network [14,23].

### 2.3. circRNAs

Different from miRNAs and lncRNAs with linear structure, circRNAs are a group of ncRNAs with a covalently closed continuous loop. Because of their loop structure, circRNAs are resistant to exonuclease-mediated degradation, and, therefore, are more stable as a biomarker than the linear lncRNAs and miRNAs [24,25]. Computationally identifying circRNAs from RNA-sequencing data and then validating by divergent-primer PCR are primary strategies for studying circRNA expression [24]. Currently, massive circRNAs have been detected in various tissue types including the human brain [24,26]. It has now been found that circRNAs are highly abundant, conserved, and dynamically expressed in the mammalian brain [26,27], which indicates their important roles in brain function and disease. Functionally, circRNAs have been found to be able to act as miRNA sponges, which contain multiple complementary binding sites to one or several different miRNAs, and play a general role in post-transcriptional gene regulation [28,29]. However, for the majority of circRNAs, their functions remain to be determined.

## 3. NcRNAs in Normal Brain Function

NcRNAs are expressed in precise regional, cellular, subcellular, and temporal patterns in developing and adult brains, which reflects their widespread and diverse influences in normal brain function. While most studies have focused on defining neurobiological roles for miRNAs, recent studies have also begun to characterize the expression and function of lncRNAs and circRNAs.

### 3.1. miRNAs in Brain Development and Function

The deletion of Dicer in the developing cerebral cortex led to the reduction of specific miRNA, which caused apoptosis in newborn neurons, a thinner cerebral cortex, and a reduction of the dendritic branch, which suggests the important roles of miRNAs in brain development. For example, the brain-enriched miR-124 was initiated at embryo day 13 (E13) and remained highly expressed throughout adulthood. It could promote the differentiation of neural progenitor cells into neurons, and be inhibited by a repressor element 1-silencing transcription factor (REST) [30]. Therefore, the REST-miR-124 axis played an important role in controlling the neuronal phenotype. More in-depth and comprehensive discussions of miRNAs on brain development can be found in previous papers [23,24].

### 3.2. lncRNAs in Brain Development and Function

The role of lncRNAs in brain development and function has been extensively studied [9,27]. Among the research, the well-studied functional lncRNA in regulating brain development included Rhabdomyosarcoma 2-associated transcript (RMST), which was regulated by the transcription factor REST and indispensable for neurogenesis. RMST was specific to the brain, and highly expressed during dopaminergic neuronal differentiation [31]. At the same time, RMST could physically interact with SOX2, which is a transcription factor known to regulate neural fate, and was required for the binding of SOX2 to promoter regions of neurogenic transcription factors. RMST and SOX2 co-regulated a large pool of downstream genes implicated in neurogenesis [32]. These findings support the role of RMST as a transcriptional co-regulator of SOX2 and a key player in the regulation of the neural stem cell fate.

### 3.3. circRNAs in Brain Development and Function

Massive studies have found that circRNAs were not only enriched in the brain, but also differentially expressed in various brain regions, which indicates their potential role in brain development [33,34,35]. The most well studied functional circRNA in the brain was CDR1as. It could serve as a trans-regulator of CDR1 mRNA through AGO2-mediated and miR-67-mediated cleavage [36]. At the same time, CDR1as could act as the sponge of miR-7, and, hence, regulated neural gene expression [37]. Another well-studied circRNA in the brain was circHomer1_a, which was derived from a linear transcript of the Homer1 gene, and could compete with the biogenesis of Homer1b/c mRNA [38]. circHomer1_a was found to regulate the synapse, the presynaptic active zone, the presynaptic membrane, and postsynaptic density [39]. For the majority of circRNAs identified, however, their functions and diverse mechanisms are not well understood yet.

## 4. NcRNAs in the Brain Tumor

There has been increasing evidence showing that ncRNAs, including miRNAs, lncRNAs, and circRNAs, play important roles in regulating almost every aspect of tumor malignancy in glioma, including tumor cell apoptosis, migration, and invasion. They may also be clinically exploited for diagnostic, prognostic, and therapeutic biomarkers.

### 4.1. miRNAs in Glioma

**Figure 2:**
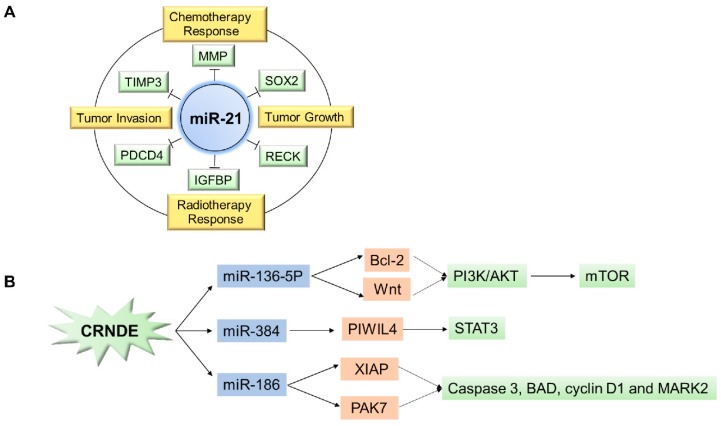
The roles of ncRNAs (miRNA, lncRNA, and circRNA) in regulating glioma biological behavior. (**A)** Functional role of miR-21 in glioma; (**B**) Functional role of lncRNA CRNDE in glioma.

A large number of oncogenic or tumor-suppressive miRNAs have been discovered and reviewed in malignant glioma during the past decade [40,41,42,43]. Among them, miR-21 was the first identified and also the most well-studied candidate [44,45]. MiR-21 was an oncogenic miRNA and had a significant upregulation in glioma when compared with the normal brain tissues. Knocking down miR-21 in malignant glioma cells would lead to inhibited cell growth, decreased invasiveness, suppressed tumorigenicity, and enhanced apoptosis both in vitro and in vivo [46,47,48,49]. Regarding the mechanism of action, miR-21 could effect a variety of cellular and molecular pathways such as insulin-like growth factor (IGF)-binding protein-3 (IGFBP3) RECK, TIMP3, MMP, and PDCD4 [46,47,49]. It was also found to be associated with stemness regulator Sox2, and can delineate glioblastoma subtypes with a prognostic impact [50]. The illustration of the functional mechanism of miR-21 in glioma is depicted in Figure 2A.

As illustrated in Figure 2A, miR-21 has multiple target genes, e.g., MMP, TIMP3, and SOX2 (depicted in a green quadrilateral). Together with these target genes, miR-21 has been reported to play key roles in regulating various traits of glioma, such as the glioma chemotherapy response, the radiotherapy response, tumor invasion, and growth (depicted in a yellow quadrilateral). As illustrated in Figure 2B, lncRNA CRNDE can interact with multiple miRNAs (depicted in a blue quadrilateral). Together with the target genes of these miRNAs (depicted in an orange quadrilateral), they are involved in various signaling pathways in glioma (depicted in a green quadrilateral).

For some other prominent miRNAs in glioma, miR-124 and miR-137 were found to be expressed at significantly lower levels in GBM tumors relative to non-neoplastic brain tissue, and were up-regulated during brain tumor stem cells differentiation [51]. Further analysis revealed that miR-124 and miR-137 could inhibit proliferation of glioblastoma multiform cells and induce differentiation of brain tumor stem cells by targeting CDK6 [51]. Moreover, miR-137 could also inhibit growth of glioblastoma through EGFR suppression [52]. For miR-124, it could act as a tumor suppressor in malignant glioma by inhibiting cell migration, invasion, stemness, and re-sensitized glioma cells to radiotherapy through various pathways. For example, miR-124 could inhibit the growth of glioblastoma by downregulating SOS1 [43], radio-sensitizing human glioma cells by targeting CDK4 [53], and suppressing the migration and invasion of glioma cells via Capn4 [54] and ROCK1 [55]. A summary of the prominent miRNA candidates in glioma is listed in Table 2.

### 4.2. lncRNAs in Glioma

Multiple lncRNAs have been reported to be involved in the initiation and progression of glioma [26,29], which include CRNDE, H19 and XIST, GAS, Malat1, Hotair, and SOX2ot. Their aberrant expression, regulatory function, and mechanism in glioma have been repeatedly revealed. Furthermore, CRNDE was extremely highly expressed in glioma as compared with normal brain tissue, and positively correlated with the tumor malignancy grade [56]. Further study revealed that CRNDE played an oncogenic role in glioma by regulating the proliferative, migratory, and invasive capacities of glioma cells. For example, CRNDE can promote glioma progression by attenuating the miR-384/PIWIL4/STAT3 axis [57].

There was a binding region between CRNDE and miR-384, and the restoration of miR-384 could suppress CRNDE expression and exert tumor-suppressive functions in glioma [57]. CRNDE could also affect the malignant biological characteristics of human glioma stem cells (GSCs) by negatively regulating miR-186 [58]. It was found that CRNDE expression was relatively down-regulated in GSCs. Overexpression of CRNDE could promote the cellular proliferation, migration, invasion, and inhibit the apoptosis of GSCs. Further investigation revealed that CRNDE decreased the expression levels of XIAP and PAK7 by binding and regulating miR-186 [58]. More recently, it was interesting to find that CRNDE actually could act as a ceRNA and regulate the interaction between miRNA and mRNA. For example, Li et al. reported that CRNDE could function as a ceRNA that bound to and negatively regulated miR-136-5p, which protected Bcl-2 and Wnt2 from miR-136-5p-mediated inhibition in glioma. This underlay the pro-tumoral actions of CRNDE [59]. All these results together suggest that CRNDE may be a key and fundamental oncogenic lncRNA in glioma. Its reported functional mechanisms in glioma is summarized and depicted in Figure 2B.

Another featured lncRNA in glioma is H19. It could be directly induced by the c-Myc oncogene to potentiate tumorigenesis [60]. Furthermore, H19 could serve as a miRNA precursor and modulate glioma progression by generating miR-675 [61], which could regulate the proliferation and migration of glioma cells by inhibiting the expression of CDK6. The knockdown of H19 suppressed tumorigenicity and stemness in U251 and U87MG glioma cells [62]. XIST was also an oncogenic lncRNA in glioma, which could promote glioma tumorigenicity and angiogenesis by acting as a molecular sponge of miR-429 [63] and maintenance of GSCs via miR-152 [64]. For the GAS5 [65], Malat1 [66,67], Hotair [68,69], and SOX2ot [70], their reported functions and mechanisms are listed in Table 2.

### 4.3. Circular RNAs in Glioma

As a newly emerging regulatory RNA class, an increasing number of studies have found that circRNAs were aberrantly expressed in gliomas, and regulated the occurrence, proliferation, migration, and invasion of the tumor [71,72,73,74,75,76]. They were also reported as potential biomarkers for the diagnosis and prognosis of glioma [71] (Table 1). For example, circRNA cZNF292 was recently identified as an angiogenesis circRNA in glioma cells. Silencing the expression of cZNF292 would arrest the cell cycle at the S/G2/M phase via the Wnt/β-catenin signaling pathway, which suppressed the glioblastoma cell proliferation and tube formation [76]. CircSMARCA5 inhibited the migration of malignant glioma cells by regulating a molecular axis involving splicing factors SRSF1/SRSF3/PTB [74]. Mechanically, circRNAs may also serve as a ceRNA or a molecular sponge to interact with miRNA, and then regulated the associated signaling pathways. For example, circTTBK2 was found to be up-regulated in glioma, and could act as the miR-217 sponge and promote cell proliferation, migration, and invasion by the miR-217/HNF1β/Derlin-1 pathway [75]. CircSHKBP1 regulated the angiogenesis of malignant glioma by interacting with miR-544a and miR-379 pathways [77]. CircNT5E could act as a sponge of miR-422a and controlled multiple pathologic processes in glioblastoma tumorigenesis, including cell proliferation, migration, and invasion [78]. CircNFIX could regulate the Notch signaling pathway to promote glioma progression by sponging miR-34a-5p [79]. Recent advances revealed that circRNA could not only act as an ncRNA, but also work like mRNA and encode protein to regulate the cellular processes in normal brain tissue and glioma. For example, circFBXW7 could encode a novel protein FBXW7-185aa, which could functionally inhibit cell proliferation. This suggests the multifaceted function of circRNAs in human cancer [73]. All these results warrant the further investigation of circRNAs in human function and disease.

## 5. NcRNAs in Exosomes and Extracellular Vesicles in Glioma

Exosomes, which are nano-sized extracellular vesicles that are secreted by most host cells, contain an abundant cargo of different RNA species and other bioactive molecules [84]. Recent advances in extracellular vesicles related to glioma revealed that exosomes secreted by tumor cells were critical mediators of intercellular communication in tumor microenvironments, and could even be used as circulating biomarkers for disease diagnosis and prognostication [85,86]. For example, the exosome levels of miR-21 from cerebrospinal fluids were associated with poor prognosis and tumor recurrence of glioma patients [80]. Furthermore, miR-148a delivered by exosomes may promote cancer cell proliferation and metastasis by targeting CADM1 to activate STAT3 pathway, which suggests a predictor and therapeutic target role of exosomal miR-148a in glioma patients [81]. For lncRNAs, exosomes from A172 glioma cell lines expressed a high level of PU03F3, which could lead to an increase in cell proliferation, migration, tube formation, and in vivo angiogenesis [82]. Using similar methods, the same research group demonstrated that the exosomal lncRNA CCAT2 released by glioma cells could enhance angiogenesis and inhibit endothelial cell apoptosis [83]. For the exosome circRNAs, it has now been revealed that circRNAs were enriched and stable in human serum exosomes. Additionally, circRNAs originated from human cancer xenografts could enter the circulation and be readily measured in the serum. Moreover, the expression levels of serum exosomal circRNAs were able to distinguish patients with colon cancer from healthy controls [87], which suggests circRNAs as a promising biomarker for cancer diagnosis. However, in glioma, no exosomal circRNAs were reported so far. The exosomal ncRNAs reported in glioma are summarized in Table 2.

## 6. NcRNAs in Neuropsychiatric Disorders

### 6.1. ncRNAs in Alzheimer’s Disease (AD)

A series of ncRNAs have been reported to be involved in the pathogenesis of AD. They played important roles in amyloid beta deposition and Tau phosphorylation, which are two key pathological characters in the pathological progression of AD. For example, miR-107 may accelerate AD progression by regulating a β-Site amyloid precursor protein-cleaving enzyme (BACE) [88]. Loss of miR-29a/b-1 cluster in sporadic AD correlated with increased BACE1/beta-secretase expression [89]. miR-9 and -181c could be down-regulated by Aβ in hippocampal cultures [90], miR-16 regulated cell death in AD by targeting amyloid precursor protein (APP) [91], and miR-124 acted as a target for BACE1 [92]. It was even found that the circulating miR-125b in patient serum could act as a diagnostic biomarker for AD [93]. As for lncRNAs, the best-studied lncRNAs in AD were BACE1-AS and BC200. BACE1-AS (β-secretase-1 antisense RNA) was implicated in a positive feedback loop that drove the amyloid cascade progression [94]. It could increase BACE1 expression by increasing BACE1 mRNA stability and generating additional BACE1 through a post-transcriptional feed-forward mechanism, and thus raised concentrations of amyloid beta and deposition of senile plaques. BC200 expression was upregulated in AD brains, and was indicated as a biomarker of AD progression [95]. Knockdown of BC200 significantly suppressed BACE1 expression, increased cell viability, and reduced cell apoptosis in the AD model, which indicates the positive relationship of BC200 with disease severity [96]. For circRNAs, it was found that ciRS-7 could promote APP and BACE1 degradation in AD by acting as the sponge of miR-7 [97]. 

### 6.2. ncRNAs in Parkinson’s Disease (PD)

As a high prevalence neurodegenerative disorder worldwide as AD, impacts of ncRNAs on the pathogenesis and/or progression of PD have been repeatedly highlighted [28]. A series of miRNAs associated with PD-related gene regulation, including SNCA, PRKN, and PARK7, PARK8 and genes involved in neuro-inflammation, have been identified [98]. They were also regarded as biomarkers for PD diagnosis, and could be detected in peripheral blood samples and cerebrospinal fluids (CSFs) [98]. However, the results between different study teams were usually with great discrepancy and failed to prove each other, which suggests that these data need meta-analysis or further validation. For lncRNAs, NEAT1 was reported to promote autophagy in an MPTP-induced PD model by stabilizing the PINK1 protein [99], and NEAT1 knockdown could effectively alleviate dopaminergic neuronal injury in vivo [99]. LncRNA Hotair promoted the onset of PD in the MPTP-mice model by upregulating LRRK2 expression [100]. In addition, Hotair knockdown provided protection against DA neuronal apoptosis by repressing caspase 3 activity. These findings suggested that inhibition of Hotair levels was an effective disease-modifying strategy in PD [100]. LncRNA Malat1 contributed to cell apoptosis in PD by sponging miR-124 [101]. For circRNAs, there was no direct evidence supporting the regulatory role of circRNAs in PD pathogenesis at present. However, because of the importance of miR-7 in PD [102], and the significant sponging role of ciRS-7 on miR-7, it is possible that ciRS-7 may exert a regulatory role in PD as well.

### 6.3. ncRNAs in Autism

Autism is a developmental disorder that is characterized by difficulty in social interaction and communication. The role of ncRNAs in autism pathological regulation has been explored in a series of studies [103]. For miRNAs, among the various miRNA candidates reported to be associated with autism, three of them were identified in more than three independent studies (miR-23a, miR-146a, and miR-106b) [104]. The networks of genes targeted by these miRNAs have significant roles in neurotrophin signaling in autism. Genome-wide changes in lncRNAs in autism were also investigated [105]. By performing RNA sequencing for 251 post-mortem samples of frontal and temporal cortex and cerebellum from 48 individuals with autism and 49 control subjects, Neelroop N. et al. identified 60 differentially expressed lncRNAs. Furthermore, 20 of these lncRNAs (including LINC00693 and LINC00689) have been shown to interact with miRNA–protein complexes, and nine with the fragile X mental retardation protein (FMRP), whose mRNA targets were enriched in autism risk genes [105]. As for the relationship between circRNA and autism, there is no direct evidence supporting the circRNA regulation on ASD at present. However, it has been reported that the RBFOX1 splicing factor, which is a gene consistently dysregulated in the brain of autism patients, might affect transcript abundance by regulating the formation of circRNA molecules [106]. This evidence suggests the potential role of circRNAs in autism, and warrants further study.

## 7. Publicly Available Tools and Platforms for ncRNA and Human Disease Study

A series of computational tools and platforms that can be used to explore the disease-related miRNAs, lncRNAs, and circRNAs, as well as their functional mechanisms, have been reported. In order to facilitate the ncRNA study in human brain function and disease, we have made a brief summarization and description of these databases here (Table 3). Several representative ones are introduced below.

### 7.1. DeepBase

DeepBase is a platform for annotating and discovering miRNAs, lncRNAs, circRNAs, and also other ncRNA classes from next generation sequencing data (http://rna.sysu.edu.cn/deepBase/) [107,108]. It provides a set of useful tools to decode the expression patterns of various ncRNA types in 19 species including humans, and to predict their functions. It also provides functional annotations for lncRNAs based on their co-expression network with protein-coding genes. Moreover, the database provides an integrative and interactive web graphical interface to display the multidimensional data, which will facilitate the transcriptional investigation and functional discovery of ncRNAs.

### 7.2. StarBase

StarBase is an open-source platform for studying the miRNA-circRNA, miRNA-lncRNA, miRNA-mRNA, miRNA-ceRNA, and protein-RNA interaction networks from large-scale CLIP-Seq data (http://starbase.sysu.edu.cn/) [109]. It can systematically identify the RNA–RNA and protein–RNA interaction networks from 108 CLIP-Seq data sets generated by 37 independent studies. By analyzing these data, the platform can characterize and include ∼9000 miRNA-circRNA, 16,000 miRNA-pseudogenes, and 285,000 protein–RNA regulatory relationships, and provides the most comprehensive CLIP-Seq experimentally supported miRNA-mRNA and miRNA-lncRNA interaction networks to date. This platform provides a very useful tool for the exploration of ncRNA functions and their coordinated regulatory networks.

### 7.3. Circ2Traits

Circ2Traits is a comprehensive database for circRNAs potentially associated with disease and traits (http://gyanxet-beta.com/circdb/) [110]. The database identifies the interactions of circRNAs with disease-associated miRNAs, and the likelihood of a circRNA being associated with a disease is calculated. Moreover, the interaction networks between circRNA and miRNA as well as mRNA and lncRNA are constructed. Following that, gene ontology (GO) enrichment analysis on the set of mRNA genes in the miRNA-circRNA interactome of individual diseases is performed to check the enrichment of genes associated with particular biological processes. It is the first comprehensive knowledge base of potential association of circRNAs with diseases in humans.

### 7.4. ExoRBase

ExoRBase is a repository of circRNA, lncRNA, and mRNA derived from RNA-Sequence data analyses of human blood exosomes (http://www.exorbase.org/) [111]. Experimental validations from the published literature are also included. ExoRBase features the integration and visualization of RNA expression profiles based on normalized RNA-Sequence data spanning both normal individuals and patients with different diseases. The first release of exoRBase contains 58,330 circRNAs, 15,501 lncRNAs, and 18,333 mRNAs. The annotation, expression level, and possible original tissues are also provided. It will aid researchers in identifying molecular signatures in blood exosomes, and will trigger new exosomal biomarker discovery and functional implication for human diseases.

## 8. Conclusions and Future Perspectives

Since ncRNAs are increasingly recognized as critical regulators in human brain function and disease, the existing studies, as reviewed and discussed above, not only provide comprehensive insights into the function of the brain, but also help us understand how molecular dysregulation at multiple levels may lead to tumoral and neuropsychiatric disorders in the human brain. These current findings raise hope for uncovering translating ncRNAs in diagnosis, prognostication, and therapy. However, due to the high complexity of human brain function, further studies are still needed. For example, while the scope of this current review is to provide a comprehensive summary of the key findings in this area, a more in-depth discussion is needed in a future review for each of these topics. Moreover, a deeper and more advanced understanding of interactions between different classes of ncRNAs, as well as their interactions with transcription factors, chromatin-modifying enzymes, and other protein effectors are also needed, to provide essential insights into the molecular mechanisms of brain function and disease.

## Figures and Tables

**Table 1:** Non-coding RNA classes and their functions.

Non-Coding RNA Classes	Functions
Transfer RNAs (tRNAs)	Function at specific sites in the ribosome during translation, help translate mRNA into protein
Ribosomal RNAs (rRNAs)	Act as the RNA component of the ribosome, help the mRNA translation
MicroRNAs (miRNAs)	Function in RNA silencing, post-transcriptional regulation of gene expression
Piwi-interacting RNAs (piRNAs)	Regulate DNA methylation, main function in germ line cells
Small nucleolar RNAs (snoRNAs)	Guide modification of other RNAs (e.g., rRNA), rRNA processing
Long non-coding RNAs (lncRNAs)	Non-protein coding transcripts longer than 200 nucleotides, heterogeneous class of RNAs, regulation of gene transcription
Circular RNAs (circRNAs)	Covalently closed RNA rings, some have coding functions, potential gene regulators and miRNA sponges

**Table 2:** Key functional ncRNAs (miRNA, lncRNA, and circRNA) in glioma.

NcRNA	Action	Reference
**MiRNA**		
miR-21	Acts as an oncogene in glioma by targeting IGFBP3, RECK, TIMP3, MMP, and PDCD4.	[46,47,49,50]
miR-124	Acts as a tumor suppressor in glioma via targeting CDK6, SOS1, CDK4, Capn4, and ROCK1.	[51,53,54,55]
miR-137	Acts as a tumor suppressor in glioma by targeting CDK6 and EGFR.	[51,52]
**LncRNA**		
CRNDE	Acts as an oncogene in glioma via multi-faced way.	[56,57,58,59]
H19	Can be directly induced by c-Myc. Serves as precursor of miR-675. Knockdown of H19 suppresses tumorigenicity and stemness in U251 and U87MG glioma cells.	[60,61,62]
XIST	An oncogenic lncRNA in glioma, which can promote glioma tumorigenicity and angiogenesis by acting as a molecular sponge of miR-429. Maintenance of GSCs via miR-152.	[63,64]
GAS5	Exerts tumor-suppressive roles in glioma cells by targeting miR-222.	[65]
MALAT1	Correlates with the malignant status and poor prognosis in glioma. Induces chemo-resistance by suppressing miR-203.	[66,67]
Hotair	An oncogenic factor in glioma. Correlates with a poor prognosis. Critical regulator of the cell cycle.	[68,69]
SOX2ot	Knockdown of SOX2OT inhibits the malignant biological behaviors of glioblastoma stem cells by up-regulating the expression of miR-194-5p and miR-122.	[70]
**CircRNA**		
circBRAF	Negatively correlates with tumor malignancy grade; Protective effect for survival.	[71]
circFBXW7	Encodes a protein FBXW7-185aa; the upregulation of FBXW7-185aa inhibited proliferation and cell cycle acceleration of glioma cells.	[72,73]
circSMARCA5	Inhibits malignant glioma cell migration.	[74]
circTTBK2	Up regulated in glioma, and can act as an miR-217 sponge and promote cell proliferation, migration, and invasion.	[75]
cZNF292	Regulates glioma angiogenesis through a Wnt/β-catenin signaling pathway.	[76]
circSHKBP1	Regulates the angiogenesis of malignant glioma by interacting with miR-544a and miR-379.	[77]
circNT5E	Acts as a sponge of miR-422a and controls multiple pathologic processes in glioblastoma tumorigenesis, including cell proliferation, migration, and invasion.	[78]
circNFIX	Regulates the Notch signaling pathway to promote glioma progression by sponging miR-34a-5p.	[79]
**Exosome ncRNA**		
miR-21	The exosome levels of miR-21 from cerebrospinal fluids are associated with a poor prognosis and tumor recurrence of glioma patients.	[80]
miR-148a	miR-148a delivered by exosomes may promote cancer cell proliferation and metastasis by targeting CADM1 to activate the STAT3 pathway.	[81]
lncRNA PU03F3	Exosomes from A172 glioma cell lines express a high level of PU03F3, which can lead to increased cell proliferation, migration, tube formation, and in vivo angiogenesis in glioma.	[82]
lncRNA CCAT2	Released by glioma cells, this exosome ncRNA can enhance angiogenesis and inhibit endothelial cell apoptosis.	[83]

**Table 3:** An overview of main human disease-associated ncRNA databases.

Name	Description	Link
**miRNA**		
DeepBase	A database for annotating and discovering small and long ncRNAs from high-throughput deep sequencing data.	https://www.webcitation.org/5tyh2Lsae?url=http://deepbase.sysu.edu.cn/
starBase	Decoding miRNA-ceRNA, miRNA-ncRNA, and protein-RNA interaction networks from large-scale CLIP-Seq data.	http://starbase.sysu.edu.cn/
microRNA.org	A database for experimentally observed miRNA expression patterns and predicts miRNA targets and target downregulation scores.	http://www.microrna.org/microrna/getExprForm.do
miRTarBase	The experimentally validated miRNA-target interaction database.	http://mirtarbase.mbc.nctu.edu.tw/php/index.php
MSDD	A manually curated database that provides comprehensive experimentally supported associations among miRNAs, SNPs, and human diseases.	http://www.bio-bigdata.com/msdd/
mirTrans	A repository that provides comprehensive information of miRNA transcription for different cell lines.	http://mcube.nju.edu.cn/jwang/lab/soft/mirtrans/
TransmiR	A database for transcription factor (TF)-miRNA regulations, through which one can find regulatory relations between TFs and miRNAs.	http://www.cuilab.cn/transmir
Cupid	A method for simultaneous prediction of miRNA-target interactions and their mediated competing endogenous RNA (ceRNA) interactions.	http://cupidtool.sourceforge.net/
miRwalk	Aggregates and compares results from other miRNA-to-mRNA databases.	http://zmf.umm.uni-heidelberg.de/apps/zmf/mirwalk2/
IMOTA	An interactive multi-omics-tissue atlas that helps you to find out more about relationships between miRNAs, proteins, and mRNAs by using charts as filters.	https://ccb-web.cs.uni-saarland.de/imota/.
**LncRNA**		
DeepBase2.0	A platform for annotating and discovering miRNAs, lncRNAs, and circRNAs from next generation sequencing data.	http://rna.sysu.edu.cn/deepBase/
lncRNdb	The reference database for functional lncRNAs.	http://lncrnadb.com/
LncRNAWiki	A wiki-based, publicly editable, and open-content platform for community curation of human lncRNAs.	http://lncrna.big.ac.cn
NONCODE	An integrated knowledge database dedicated to ncRNAs, especially lncRNAs.	http://www.noncode.org
lncRNome	A comprehensive searchable biologically oriented knowledgebase for lncRNAs in humans.	http://genome.igib.res.in/lncRNome/
NONCODE	A systematic database that is dedicated to present the most complete collection and annotation of ncRNAs, especially lncRNAs.	http://www.bioinfo.org/noncode/
RISE	A comprehensive repository of RNA-RNA interactions involving mRNA and lncRNAs.	http://rise.life.tsinghua.edu.cn/
Lnc2Meth	A comprehensive resource and web tool for clarifying the regulatory relationships between human lncRNAs and associated DNA methylation in diverse diseases.	http://bio-bigdata.hrbmu.edu.cn/Lnc2Meth/
**circRNA**		
circRNABase	Decodes miRNA-circRNA interaction network from CLIP-Seq data.	http://starbase.sysu.edu.cn/mirCircRNA.php
circBase	Explores public circRNA datasets or discover circRNAs in your own RNA-Seq data.	http://www.circbase.org/
CircNet	A database of circRNAs derived from transcriptome sequencing data.	http://circnet.mbc.nctu.edu.tw/
Circ2Traits	A comprehensive collection for circRNAs is potentially associated with diseases and traits.	http://gyanxet-beta.com/circdb/
CSCD	A database for cancer-specific circRNAs.	http://gb.whu.edu.cn/CSCD/
**Database for Circulating ncRNAs**		
miRandola	A comprehensive manually curated classification of different extracellular circulating ncRNA types.	http://mirandola.iit.cnr.it/
ExoRBase	A repository of circRNA, lncRNA, and mRNA derived from RNA-Sequence data analyses of human blood exosomes.	http://wwww.exorbase.org
